# Investigation of Microstructure Evolution and Phase Selection of Peritectic Cuce Alloy During High-Temperature Gradient Directional Solidification

**DOI:** 10.3390/ma13040911

**Published:** 2020-02-19

**Authors:** Yiku Xu, Zhaohao Huang, Yongnan Chen, Junxia Xiao, Jianmin Hao, Xianghui Hou, Lin Liu

**Affiliations:** 1 School of Material Science and Engineering, Chang’an University, Xi’an 710064, China; 1051276179h@gmail.com (Z.H.); frank_cyn@163.com (Y.C.); ayjunxjx@163.com (J.X.); h-jianmin@126.com (J.H.); 2 Faculty of Engineering, The University of Nottingham, University Park, Nottingham NG7 2RD, UK; 3 State Key Laboratory of Solidification and Processing, Northwestern Polytechnical University, Xi’an 710072, China; linliu@nwpu.edu.cn

**Keywords:** CuCe alloy, directional solidification, primary spacing, phase selection, cellular peritectic coupled growth

## Abstract

In this work, a CuCe alloy was prepared using a directional solidification method at a series of withdrawal rates of 100, 25, 10, 8, and 5 μm/s. We found that the primary phase microstructure transforms from cellular crystals to cellular peritectic coupled growth and eventually, changes into dendrites as the withdrawal rate increases. The phase constituents in the directionally solidified samples were confirmed to be Cu_2_Ce, CuCe, and CuCe + Ce eutectics. The primary dendrite spacing was significantly refined with an increasing withdrawal rate, resulting in higher compressive strength and strain. Moreover, the cellular peritectic coupled growth at 10 μm/s further strengthened the alloy, with its compressive property reaching the maximum value of 266 MPa. Directional solidification was proven to be an impactful method to enhance the mechanical properties and produce well-aligned in situ composites in peritectic systems.

## 1. Introduction

A number of important binary alloys exhibit peritectic transitions during solidification, such as Fe-Ni, Fe-C, Cu-Zn, Ti-Al, and Sn-Ni [1,2,3,4,5,6]. In these cases, the primary solid α-phase precipitated from the liquid phase reacts with the liquid phases to form a peritectic β-phase at the peritectic temperature. Binary alloys with peritectic compositions can form a variety of microstructures in directional solidification experiments, such as discrete bands of the α-phase and β-phase, island structure, and primary cell/dendritic crystal, and cellular peritectic coupled growth (CPCG) [7,8].

The primary dendrite arm space is one of the most important microstructural parameters in the directional solidification structure and is mainly controlled by the growth conditions (thermal gradient G and withdrawal rate V) for a given alloy [9]. Larger G and V tend to make the primary dendrite arm spacing smaller. The smaller primary dendrite arm spacing represents a refined dendritic structure, which usually improves the mechanical properties of the alloy. Directionally solidified binary alloys have been extensively studied, and various models for describing the relationship between the withdrawal rate and the primary spacing have been obtained. Among them are the more commonly used Hunt model based on the Bower–Brody–Flemings model, the Trivedi model, and the Luo model for predicting the cellular spacing of CPCG [10,11,12]. These models assume that the temperature and composition distribution between the intercellular/interdendritic are uniform in a direction perpendicular to the growth direction. The shape of the tip of the cell/dendritic crystal is assumed to solve the solute field during the growth of the cell/dendritic. The Hunt model assumes that the dendrite tip is spherical, while the Trivedi model assumes it is to be a rotating paraboloid. The effect of the volume fraction of each phase on the primary spacing has been investigated in the Luo model. The details of these three models are as follows:

Hunt model [10]

(1) $$\lambda_{1}=2.83({k\Delta T}_{0}\Gamma D{)}^{\frac{1}{4}}G^{-\frac{1}{2}}V^{-\frac{1}{4}},$$

Trivedi model [11]

(2) $$\lambda_{1}=2\sqrt{2}({28k\Delta T}_{0}\Gamma D{)}^{\frac{1}{4}}G^{-\frac{1}{2}}V^{-\frac{1}{4}},$$

Luo model [12]

(3) $$\lambda_{1}=2.84{\left(\frac{\Delta T^{\prime }\left(1+\xi \right)}{\xi }\right)}^{\frac{1}{2}}{\left(\frac{D\Gamma }{k\Delta T_{0}}\right)}^{\frac{1}{4}}G^{-\frac{1}{2}}V^{-\frac{1}{4}}.$$

The Cu-Ce alloys have attracted attention since Hanaman first drew a phase diagram of thermal analysis for studying the glass-forming region in metallic glass [13,14]. The addition of Cu-Ce alloy can effectively improve the performance of iron-based metal bonds [15]. Our previous study confirmed that an interesting island structure of Cu-21 wt% Ce alloy can be formed during directional solidification, which involves island-like primary phases dispersed in the peritectic phase matrix [16]. As a typical peritectic composition of CuCe alloy, recent research has proved that, as an additive, it can effectively improve the performance of Ni-Cr alloy brazed diamond [17].

In this paper, CuCe alloys were prepared at different withdrawal rates through high-temperature gradient directional solidification (DS) method. The microstructure evolution and phase selection were investigated through optical microscopy (OM), scanning electron microscopy (SEM), and transmission electron microscopy (TEM). The primary dendrite arm spacing of the peritectic CuCe alloy was discussed based on both the experimental and simulation result. The compressive properties of Cu-Ce alloys were further studied corresponding to different withdrawal rates and the relating peritectic directional solidified structures.

## 2. Experimental

In this study, a peritectic CuCe alloy was selected with a nominal composition of 50 at.% Cu and 50 at.% Ce. Metal sheets of 99.95 wt% Cu and metal blocks of 99.9 wt% Ce were rubbed using sandpaper in an argon glove box and then ultrasonically cleaned to get rid of the external oxide layer. CuCe alloys with the proper weight of Cu sheet and Ce block were prepared and melted using a vacuum arc melting furnace under Zr-gettered Ar atmosphere to investigate the arc melting behavior. The buttons were turned 180° and remelted several times to ensure homogeneity.

For the directional solidification process, raw materials with the appropriate mass were prepared for melting using a vacuum induction furnace (ZG-0.025) (Xinhuadi Metallurgical Equipment Manufacturing Co., Ltd., Jinzhou, China), and CuCe ingots were cast into shapes, 30 mm in diameter and 85 mm in length. It was necessary to discharge oxygen with argon and maintain a high degree of vacuum in the smelting process, due to the high oxidation rate of Ce [18]. Cylindrical rods, 7 mm in diameter and 85 mm in length, were prepared for directional solidification using a wire-electrode cutting machine. A Bridgman induction furnace was applied for directional solidification experiments, which was designed by the State Key Laboratory of Solidification Technology of the Northwestern Polytechnical University. The temperature gradients were measured to be 200 K/cm using a thermocouple.

The metallographic samples were sanded with 200 to 2000 mesh SiC sandpaper, and then polished with Al2O3 powder for microscopic observation. The microstructures of the samples were investigated by optical microscopy of an Axio Scope-A1 (Zeiss, Jena, Germany) equipped with a Carl Zeiss digital camera and scanning electron microscope (S-4800) (Hitachi, Tokyo, Japan). The phase composition of the alloy was analyzed by X-ray diffraction (XRD) (Bruker D8 Advance model) using Cu–Kα radiation scanning from 20° to 100° at a scanning rate of 8° min^−1^. The samples were processed to below 100 nm using an ion thinner, and then observed with a transmission electron microscopy (FEI Talos F200X TEM) (FEI, Hillsboro, OR, USA). Compositional mapping was analyzed using energy dispersive spectrometry (EDS) affiliated with SEM and TEM. The transition temperature of the CuCe alloy was measured by differential scanning calorimetry (DSC) (STD650) (TA Instruments, Newcastle, WA, USA). Quasi-static compression tests were conducted in MTS, up to 20 kN loading, which achieved a deformation rate of 5 mm/min. A Vickers hardness tester (HX-1000TM) (Shanghai Optical Instrument Factory, Shanghai, China) was employed to determine the microhardness using a load of 100 g. Table 1 shows the parameters used in this study.

## 3. Results and Discussion

### 3.1. Directional Solidified Microstructure of CuCe Alloys

#### 3.1.1. Microstructure Evolution of the CuCe Alloy

**Figure 1:**
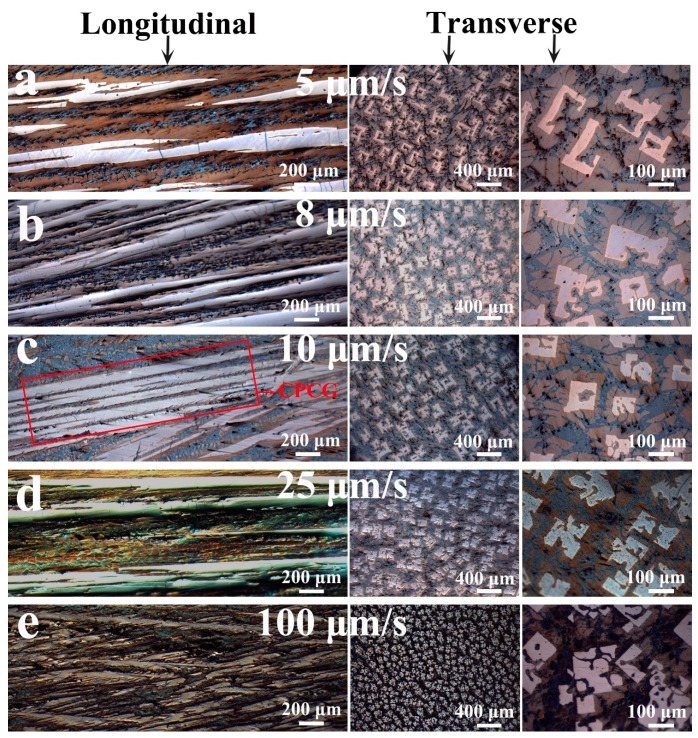
Optical microscopy (OM) images of CuCe alloys at varying withdrawal rates. (**a**) 5 μm/s, (**b**) 8 μm/s, (**c**) 10 μm/s, (**d**) 25 μm/s, and (**e**) 100 μm/s.

The optical microscopy images of the cross-section and longitudinal section of the directional solidification stabilization stage of the CuCe alloys are shown in Figure 1, which indicate the structures primarily consisting of the primary dendritic/cellular phase and the interdendritic phase. Figure 1a,b shows that the dendrites become dissolved and the number of primary phase grains in the transverse image is relatively small. Regularly arranged primary dendrite phases can be observed in Figure 1c. It is interesting that the dendritic crystals in Figure 1d have a clear orientation and are arranged neatly, comparing with the tilted dendrites in Figure 1e. Under equilibrium solidification, the CuCe alloy underwent three reactions [21]:

(4) $$L\overset{692{}^{\circ}C}{\to }{\mathrm{Cu}}_{2}\mathrm{Ce}+L$$

(5) $$L+{\mathrm{Cu}}_{2}\mathrm{Ce}\overset{492{}^{\circ}C}{\to }\mathrm{CuCe}+L$$

(6) $$L\overset{407{}^{\circ}C}{\to }\mathrm{CuCe}+\mathrm{Ce}.$$

In Figure 1a–e, at V ≤ 10 μm/s, the low withdrawal rate and high temperature related to low G/V and constitutional supercooling, along with distinct cellular growth characteristics are observed. Meanwhile, it can be seen from the longitudinal section that the cell crystals begin to become discontinuous as the withdrawal rate decreases. When V = 5 μm/s, the primary phase has been packaged by the peritectic phase to form intermittent cell crystals. As the withdrawal rate increases, the solidified morphology is transformed from cellular to dendritic. As V ≥ 25 μm/s, the increased constitutional supercooling develops a discontinuous dendritic crystal. In summary, as the withdrawal rate increases from 5 to 100 μm/s, the directional solidification microstructure changes as follows: intermittent cellular crystal → cellular crystal → dendritic crystal.

At a withdrawal rate of 5 μm/s, the peritectic reaction caused the cell crystals to become intermittent. A low withdrawal rate allows for sufficient time for the peritectic reaction to dissolve the primary phase dendrites, causing the dendrites to become discontinuous. This is a transitional structure that transforms from cellular into an island band. This structural formation is caused by the different growth rates of the primary phase and the peritectic phase. The concave liquid/primary phase interfaces, near the liquid/primary phase/peritectic phase trijunctions, decrease the local growth speed of the primary phase near the trijunctions, whereas the peritectic reaction enhances the growth rate of the peritectic phase [22]. That is to say, the peritectic reaction develops the peritectic phase to fully engulf the primary phase, thus increasing the tendency to form an island banding structure.

**Figure 2:**
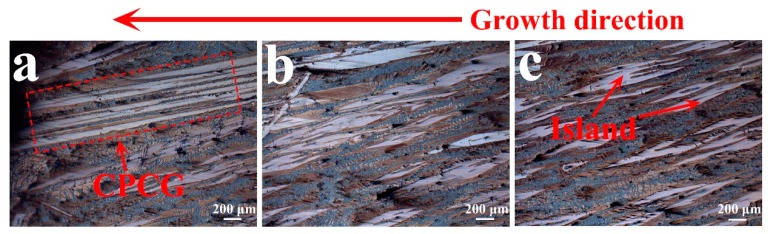
The Optical microscope (OM) image of the longitudinal section of the CuCe alloy at a withdrawal rate of 10 μm/s. (**a**) Cellular peritectic coupled growth (CPCG); (**b**) Closed island structure, and (**c**) Island structure.

It can be seen from Figure 2 that when the withdrawal rate is 10 μm/s, the transition from island to cellular peritectic coupled growth occurs in the directionally solidified structure. The same structural transformation was also found in the study of Fe-Ni alloys [12]. The present experiments definitely prove that island banding is an initiating mode for coupled growth in the peritectic phase. The rows of aligned islands supply a roughly periodic initial condition for fingers of the matrix of one phase to emerge in the space between the islands of the other phase, which is conductive to the growth of a coupled growth structure.

#### 3.1.2. Grain Size and Phase Volume Fraction

The cross-section was chosen to calculate the columnar grain size (d) of the primary phase [23]. The average area (a) of the grains was calculated by determining the number of grains (N) in the specified area (A):

*a* = *A*/*N.* (7)

Because the cell/dendritic is usually considered a cylinder, the grains were assumed to be round [10]. The average grain size (*d*) was calculated by [23]:

*a* = π(*d*/2)^2^. (8)

As Table 2 indicated, the obtained grain size of the primary phase reduced with an increase of the withdrawal rate, a maximum value of 156.148 μm presenting at 5 μm/s.

Irregularity is known to be one characteristic of the microstructure in common materials, while the microstructure is usually assumed to be a repetitive pattern in stereological analysis. The use of fractal geometry in the analysis of irregular features has become an aid in developing a better understanding of the relationships among different fields of knowledge [24]. In this work, the fractal dimensions at different withdrawal rates were calculated by MATLAB through averaging 30 primary phase grains. The result showed the fractal dimension of the primary phase grains decreased from 1.7487 at 100 μm/s to 1.5823 at 5 μm/s. A lower fractal dimension produces a more complex graphics surface. The peritectic reaction will dissolve the primary phase, causing holes to form in the new surface, and the surface will become uneven. Therefore, the decrease of fractal dimension indicates that the peritectic reaction becomes more sufficient as the withdrawal rate increases. The fractal dimension of the primary phase grains is proved to increase with the reduction in grain size.

#### 3.1.3. Volume Fraction of Each Phase

**Figure 3:**
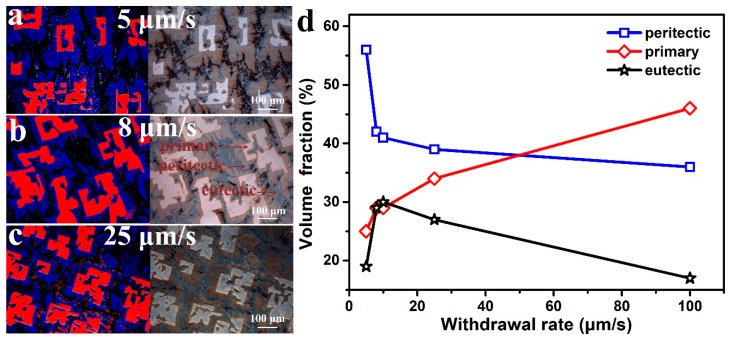
The CuCe alloy transverse microstructure image and staining picture. (**a**) 5 μm/s, (**b**) 8 μm/s, (**c**) 25 μm/s. (**d**) Volume fraction of each phase.

Different directionally solidified phases at different withdrawal rates are demonstrated in Figure 3. Figure 3d reveals the volume fraction of three different phases calculated by the Image-Pro Plus software from the OM photos. As the withdrawal rates increased from 5 to 25 μm/s, the peritectic phase clearly decreased. At the same time, the volume fraction of the primary phase also increased as the withdrawal rate increased. Furthermore, the thickness of the primary phase gradually decreased, and more dendrites were developed with increasing withdrawal rates. A short peritectic reaction time led to non-equilibrium solidification during the experimental process, and as a result, the peritectic phase cannot be fully extended. A thin peritectic phase can be seen as no sufficient growth occurs for the high withdrawal rate. The peritectic phase became thinner as the withdrawal rate increased. A significant amount of data was used to test the phase ratio of the peritectic CuCe alloy at different withdrawal rates, as listed in Figure 3. Clearly, the primary phase decreased with the increase of the withdrawal rate for a given alloy. At 5 μm/s, the ratio of the peritectic phase is larger than the others. The withdrawal rate has a great influence on the peritectic reaction, where a lower withdrawal rate was beneficial to the peritectic reaction. At a lower withdrawal rate, the highest peritectic phase and lowest primary phase were obtained.

### 3.2. Primary Spacing

#### 3.2.1. Measurement of Primary Spacing

The primary dendritic spacing is usually measured by two methods. One is the area method [25], where the number of primary dendrites in an area of *A* is *N*, and the primary dendritic spacing is calculated as follows [25]:

(9) λ1=k′(A/N)12

where λ_1_ is the primary dendritic spacing and *k*′ is a constant related to the arrangement of the primary dendrites. The other is the minimum spanning tree (MST) method, which is a connected graph containing all cell crystals/dendrites without closed loops and ensures a minimum sum of side lengths [26]. Comparing the two methods, MST can reduce human error and increase the sample size, so the MST method should have higher accuracy [27].

#### 3.2.2. Calculation of the Primary Dendrite Spacing with Several Models

An unstable solid/liquid interface leads to a cellular microstructure. The cellular microstructure appeared at a withdrawal rate of lower than 10 μm/s. The interface was more unstable, and the preferred orientation appeared along the heat flow direction with increasing withdrawal rates, which finally transferred into dendrites. The characteristic length of dendrites can be governed by adjusting the solidification parameters.

**Figure 4:**
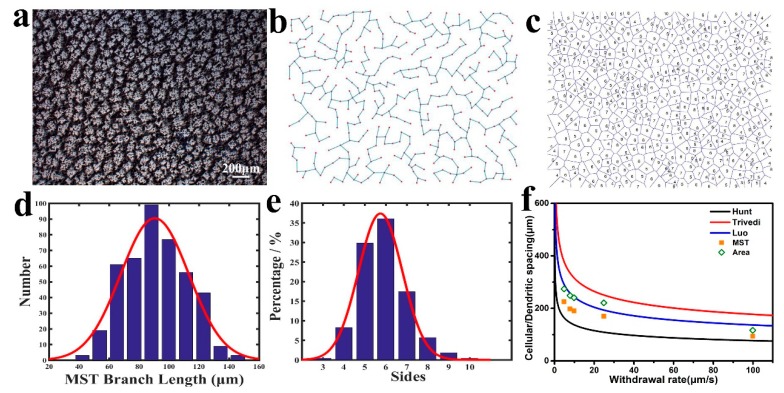
Statistical analysis of cell/dendrite distribution characteristics (**a**) Cross-section structure at a withdrawal rate of 100 μm/s. (**b**) The minimum spanning tree corresponding to (**a**). (**c**) Voronoi polygon form of (**a**). (**d**,**e**) Gaussian distribution of minimum spanning tree (MST) and Voronoi polygon statistics. (**f**) Comparison of the experimental dendritic arm spacing and theoretical model.

Figure 4b,c are an MST diagram and a Voronoi polygon diagram obtained according to Figure 4a. A Voronoi polygon refers to the nearest neighbor cell number of each dendrite/cell crystal and is widely used to analyze the distribution law of cells. In Figure 4d, λ is the calculation result corresponding to Figure 4b,c, and the red curve in the figure is a Gaussian distribution curve. The primary dendrite spacing and the nearest neighboring dendrite have a typical Gaussian distribution with fitting coefficients, R^2^, of 0.941 and 0.988, respectively. Figure 4f is a comparison of the experimental dendritic spacing with various models. It can be seen from the figure that the primary dendrite/cell spacing increases with a decreasing withdrawal rate, reaching a minimum of 93.875 μm at 100 μm/s. The primary dendrite spacing obtained by the area method is larger than the MST method. In this experiment, the results have the same trend as the Hunt, Luo, and Trivedi models, but they are not completely consistent with these models.

The results of the Hunt and Trivedi models have a large deviation from the experimental data. However, the statistically obtained primary spacing is closer to the Luo model. The different results of these three theoretical models may be caused by different starting points and approximate assumptions. The Burden–Hunt model was used to calculate the steady-state diffusion field in the Hunt model to obtain the relationship between the tip super-cooling degree and λ_1_. The main drawback of the Burden–Hunt model was the assumption that the radius of the dendrite had no influence on the temperature gradient, and the effect of the second phase volume fraction on the dendrite spacing was not considered. In contrast, the Luo model that is influenced by the second phase volume fraction is closer to the experimental results.

### 3.3. Phase Selection

**Figure 5:**
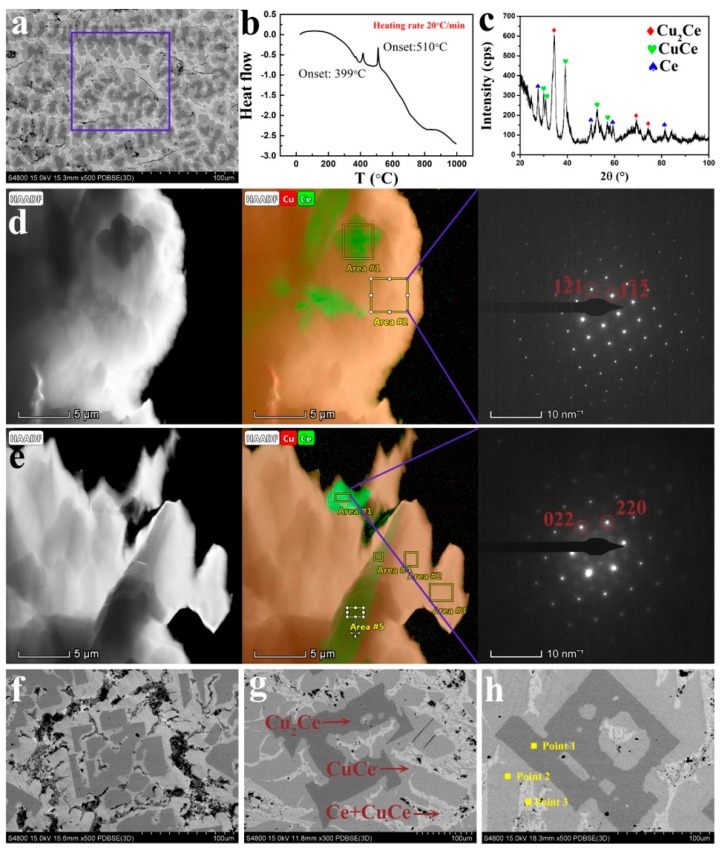
(**a**) Scanning electron microscopy (SEM) image of casting Cu-Ce alloy, (**b**) differential scanning calorimetry (DSC) curve of a Cu-Ce alloy, (**c**) X-ray diffraction spectrum of casting Cu-Ce alloy. (**d**,**e**) Transmission electron microscopy (TEM) dark field image of Cu-Ce alloy, corresponding energy dispersive spectrometry (EDS) and selected area electron diffraction pattern. (**f**–**h**) SEM images with a withdrawal rate of 100, 25, and 10 μm/s.

In Figure 5a, the composition of the as-cast CuCe alloy (51.6 ± 0.5) at.% Cu, and (48.4 ± 0.5) at.% Ce was determined by an EDS area scan. It was determined that the alloy is slightly Ce-depleted compared to the nominal stoichiometry due to processing losses. Phase transformation peaks occurred at 399 °C and 510 °C, shown in the DSC curve, which correlated well with the phase diagram. In Figure 5c, the crystal phases of Cu_2_Ce, CuCe, and Ce were further confirmed by x-ray diffraction. The position of the diffraction peak and the relative intensity are in good agreement with the standard data.

It can be found in Figure 5d,e that a bulk structure distributed on the matrix. Table 3 shows detailed EDS data corresponding to the TEM images. The EDS data shows the average of the Cu element and the Ce element content in area 1 in Figure 5d and areas 4 and 5 in Figure 5e, which are 47.76 at.% and 52.24 at.%, respectively. The average content of Cu and Ce in the yellow area of the EDS mapping in Figure 5d,e is 69.74 at.% and 30.26 at.%, respectively. The content of Cu and Ce in region 1 of the EDS mapping in Figure 5e is 11.16 at.% and 88.84 at.%, respectively. When the EDS is tested, a copper mesh supporting the transmission sample is scanned, so the content of Cu in the EDS data is high. As shown, the atomic ratio results of Cu and Ce elements are about 2:1, 1:1, and 0:1 respectively, indicating that the existing phases might be Cu_2_Ce, CuCe, and Ce. The selected area electron diffraction patterns from Figure 5d,e were determined to be the Cu_2_Ce and Ce phases.

SEM microstructures (Figure 5) can roughly estimate the volume fractions of two phases in the CuCe alloy. However, the volume fraction observed here somewhat deviates from the theory. In clarifying the phases of the black (region A in Figure 5g), gray (region B in Figure 5g), and white regions (region C in Figure 5g), combined with the results of EDS, XRD, and SAED, it was found that the cerium content increased from black to white as the withdrawal rates increased from 10 to 100 μm/s in the alloy. The black region is mainly the Cu_2_Ce phase, the gray is the CuCe phase, and the white is the Ce + CuCe eutectic structure. The above analysis reveals that the microstructure is the Cu_2_Ce phase, CuCe phase, and Ce phase, which differ from the equilibrium solidification. According to the above analysis, the primary phase is in the black region and the peritectic phase is in the gray region in Figure 5. The peritectic phase completely separates the primary phase from the base phase.

### 3.4. Mechanical Properties Characterization

**Figure 6:**
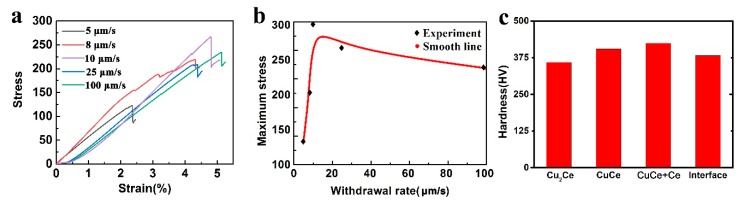
(**a**) The compressive strain–stress curves of directionally solidified CuCe alloy; (**b**) The relationship between the withdrawal rate and the maximum stress; (**c**) The microhardness of different phases.

The compressive properties of the directionally solidified samples with a Φ 7 × 15 (mm) shape were investigated, at a compression rate of 5 mm/min. No obvious yielding curves can be seen from these five withdrawal rates in the room-temperature compressive strain–stress curves (Figure 6a). All five compression curves had both linear elastic and plastic collapse stages. Elastic deformation occurred at the beginning of the compression when the stress and strain showed a linear relationship. Different withdrawal rates led to different maximum compressive forces. After reaching the maximum compressive force, the sample crushing stopped. Referring to the values of the stress, these change with the strain of the samples, as shown in Figure 6a. Table 4 exhibits the detailed mechanical properties at room temperature.

The relationship between the withdrawal rate and the maximum stress is displayed in Figure 6b. As the withdrawal rate increases, the compressive stress and compressive strain generally increase. It is worth noting that the compressive stress reaches a maximum of 266 MPa at 10 μm/s, and the compressive strain reaches a high value of 4.83%. This improvement in mechanical properties may be due to several reasons. As discussed, the fractal dimension gradually increases with the increase of the withdrawal rate, indicating the surface of the primary dendritic phase is smoother and more regular. It has been reported that with the increase of the drawing rate, the fluctuation of the number of nearest neighbor dendrites of a single dendrite is smaller, closer to seven [16]. This shows that the distribution of the dendrite array is more uniform. The increasing withdrawal rate can reduce the primary dendrite arm spacing. Adjacent dendrites with more regular shapes become closer together, resulting in the entire refined dendrite array. Dislocations tend to accumulate on the slip surface of the refined dendrite array. Therefore, alloys at higher drawing rates have better compression properties due to the restricted dislocation movement. Cruz believes that the strength of the alloy is related to the distribution of the dendrite array, the second phase distribution, and the dislocations slip. The improvement in alloy strength is due to the refinement of the dendrite array. The shorter wavelength of the periodicity of the microsegregation results in better mechanical properties of castings with smaller dendrite spacings [28]. The research of Hernando confirms that refinement of primary austenite can improve the tensile properties of Fe-Si-C alloys [29]. In addition, Campbell pointed out that the decrease in the dendrite spacing makes the interdendritic structure more uniform, clearer, and stronger, which improves the properties [30].

The peak of the strain that occurs at 10 μm/s may be due to the appearance of CPCG. According to Campbell’s report, more uniform, regular dendrites and interdendritic structures will have better mechanical properties. The coupled growth results in the primary phase and the peritectic phase being alternately distributed periodically, thus leading to improved mechanical properties. The effect of this structure on properties is also widely found in other alloys, such as the Al-Ni alloy [31], AZ31 magnesium alloy [32], and Al0.7CoCrFeNi high-entropy alloy [33].

Directional solidification is an effective means to improve the compression properties of CuCe alloy. Figure 6c showed the microhardness of the different phases of the CuCe alloy. The hardness of the CuCe + Ce eutectic structure was up to 424.38 HV. With a decrease in Ce content, the hardness gradually decreased to 359.14 HV for Cu_2_Ce. The hardness at the interface between the CuCe and Cu_2_Ce phases was 382.57 HV. In general, the hardness of a material is related to its crystal structure. Close-packed structures have higher hardness than other structures. The hardness of the Ce face-centered cubic is greater than that of Cu_2_Ce and CuCe in the tetragonal system. Therefore, the hardness of the Cu+CeCu eutectic structure is greater than that of other phases.

## 4. Conclusions

A CuCe alloy was prepared by directional solidification at various withdrawal rates (100, 25, 10, 8, and 5 μm/s). Our specific conclusions are given as follows:

(a) The microstructure was transformed from intermittent cellular crystals to cellular crystals, and finally, to dendritic crystals as the withdrawal rate decreased from 100 to 5 μm/s. Cellular peritectic coupling growth was found at 10 μm/s. As the withdrawal rate decreased, the average size of the primary phase grains gradually increased, and the fractal dimension gradually decreased.

(b) The primary dendrite/cellular spacing increased as the withdrawal rates decreased from 100 to 5 μm/s. The relationship between the primary dendrite spacing and the withdrawal rate was consistent with the predictions of the Luo model.

(c) In the directionally solidified CuCe alloy, the primary phase and the peritectic phase were Cu_2_Ce and CuCe, respectively. A Cu + CuCe eutectic structure was found.

(d) As the withdrawal rate decreased, the primary spacing gradually increased, and the compressive stress and compressive strain generally decreased. At the same time, the compressive stress and compressive stress fluctuated at 10 μm/s, which were 266 MPa and 4.83%, respectively, and the corresponding microstructure was cellular peritectic coupled growth. The experimental results show that the CuCe alloy can obtain better compression performance when cellular peritectic coupled growth occurs.

## Figures and Tables

**Table 1:** Thermophysical parameters of a peritectic CuCe alloy.

Thermophysical Parameters	Symbol	Data
Solidification rate	*V*	5, 8, 10, 25, 100 μm/s
Temperature gradient	G	200 K/cm
Equilibrium solute distribution coefficient	k	0.53
GIBBS-THOMSON coefficient	Γ	0.00001 Kcm [19]
Solute Diffusion Coefficient in Liquid Phase	D	0.00002 cm^2^/s [20]
Balance solidification crystallization temperature interval	ΔT_0_	207 K
Ratio of two-phase volume fraction	ξ	0.7058
Primary phase and cell phase flat temperature difference	ΔT ’	22.64

**Table 2:** Geometrical characteristics of the primary phase at different withdrawal rates.

Withdrawal Rate	5 μm/s	8 μm/s	10 μm/s	25 μm/s	100 μm/s
d (μm)	156.1481	144.9771	144.2626	140.4628	59.9433
Fractal Dimension	1.5823	1.7162	1.7208	1.7423	1.7487

**Table 3:** The EDS data of directionally solidified CuCe alloys.

		Cu (Atomic Fraction)	Ce (Atomic Fraction)
Figure 5d	Area 1	46.62%	53.38%
	Area 2	70.16%	29.84%
Figure 5e	Area 1	11.16%	88.84%
	Area 2	69.53%	30.47%
	Area 3	69.64%	30.36%
	Area 4	48.81%	51.19%
	Area 5	47.86%	52.14%
Figure 5h	Point 1	68.96%	31.04%
	Point 2	51.26%	48.74%
	Point 3	3.73%	96.27%

**Table 4:** The compression properties of CuCe alloy at different withdrawal rates.

Withdrawal Rate	5 μm/s	8 μm/s	10 μm/s	25 μm/s	100 μm/s
Maximum compression force (kN)	5.70	8.43	9.42	10.26	11.61
Compressive strength (MPa)	128	187	266	198	220
Elastic modulus (MPa)	3620	4540	3460	4580	4760
Compressive strain (%)	2.27	3.19	4.83	4.32	5.08
